# A Bivariate Mapping Model Identifies Major Covariation QTLs for Biomass Allocation Between Leaf and Stem Growth of *Catalpa bungei*

**DOI:** 10.3389/fgene.2021.758209

**Published:** 2021-11-18

**Authors:** Miaomiao Zhang, Nan Lu, Tianqing Zhu, Guijuan Yang, Guanzheng Qu, Chaozhong Shi, Yue Fei, Bingyang Liu, Wenjun Ma, Junhui Wang

**Affiliations:** ^1^ State Key Laboratory of Tree Genetics and Breeding, Key Laboratory of Tree Breeding and Cultivation of State Forestry Administration, Research Institute of Forestry, Chinese Academy of Forestry, Beijing, China; ^2^ State Key Laboratory of Tree Genetics and Breeding, Northeast Forestry University, Harbin, China

**Keywords:** bivariate mapping model, biomass allocation, covariation, quantitative trait locus, *Catalpa bungei*

## Abstract

Biomass allocation plays a critical role in plant morphological formation and phenotypic plasticity, which greatly impact plant adaptability and competitiveness. While empirical studies on plant biomass allocation have focused on molecular biology and ecology approaches, detailed insight into the genetic basis of biomass allocation between leaf and stem growth is still lacking. Herein, we constructed a bivariate mapping model to identify covariation QTLs governing carbon (C) allocation between the leaves and stem as well as the covariation of traits within and between organs in a full-sib mapping population of *C. bungei*. A total of 123 *cov*QTLs were detected for 23 trait pairs, including six leaf traits (leaf length, width, area, perimeter, length/width ratio and petiole length) and five stem traits (height, diameter at breast height, wood density, stemwood volume and stemwood biomass). The candidate genes were further identified in tissue-specific gene expression data, which provided insights into the genetic architecture underlying C allocation for traits or organs. The key QTLs related to growth and biomass allocation, which included *UVH1*, *CLPT2*, *GAD/SPL*, *COG1* and *MTERF4*, were characterised and verified via gene function annotation and expression profiling. The integration of a bivariate Quantitative trait locus mapping model and gene expression profiling will enable the elucidation of genetic architecture underlying biomass allocation and covariation growth, in turn providing a theoretical basis for forest molecular marker-assisted breeding with specific C allocation strategies for adaptation to heterogeneous environments.

## Introduction

Plants are organised into various organs that have distinct functional divisions and carry out continuous material exchange. Leaves are the major organs for photosynthesis, respiration and transpiration, exhibiting tremendous diversity in shape and size (Nocera, 2017). Plant stems play key roles in nutrients and water transport, while also providing physical support (Niinemets, 2007; Brienen et al., 2017). The balanced allocation of carbon (C) to various plant organs is crucial for plant growth and development, as it allows the formation of specific plant morphology as well as phenotypic plasticity, which in turn have a profound impact on plant adaptability and competitiveness under fluctuating environmental conditions (Poorter et al., 2019). The translocation of carbohydrates from the photosynthesising “source” leaves provide substrates required for the growth of non-photosynthesising “sink” organs. The plant architecture determined by stems and branches is closely associated with foliage photosynthetic efficiency, cultivation, yield, light assimilation and harvesting. C allocation patterns also emerge among different tissues and traits within a single organ, such as taproot-lateral root, leaf thickness and surface area as well as diameter at breast height (Chen et al., 2021). For instance, expression analysis in *Arabidopsis* suggested that excessive *SMAX1* expression suppressed rosette shoot branching, while promoting leaf and petiole elongation via regulating *TCP1* expression (Zheng et al., 2021). Currently, biomass allocation represents a hot topic in the fields of plant molecular biology, evolutionary genetics and ecology (Weraduwage et al., 2015; Berny Mier et al., 2019; Lauri, 2019). However, little is known regarding the genetic basis, which governs the interaction and coordination of leaf and stem growth in plants, especially in forest trees.

Quantitative trait locus (QTL) mapping is a valuable approach for exploring specific genes underlying complex traits (Miles and Wayne, 2008). This approach has been previously utilised for the study of growth-related traits in wheat (Semagn et al., 2021), maize (Guo et al., 2021), *Arabidopsis thaliana* (Getahun et al., 2020), *Populus trichocarpa* (Chhetri et al., 2019) and *Eucalyptus* (Ammitzboll et al., 2020). A reasonable trade-off strategy for C distribution among organs or phenotypes optimises the resource acquisition, viability and adaptability of plants (Poorter et al., 2012). For woody plants, Novaes et al. (2009) identified QTLs implicated in multiple traits, which regulated C allocation between plant growth and wood components (lignin and cellulose). The identification of pleiotropic QTLs can significantly improve the accuracy of genetic mapping and advance the molecular marker-assisted breeding process. Thus, introducing the concept of C allocation into the genetic framework is of great value for the elucidation of genetic mechanisms underlying phenotypic plasticity and pleiotropy. However, research on the genetic basis of plant C allocation has been is scarce due to the lack of suitable conceptual frameworks.

Bivariate trait correlations can be quantitatively expressed through statistical models in order to reveal C distribution patterns and dissect the underlying genetic basis. The first experimental utilisation of QTL mapping for the study of biomass allocation was reported by Wu et al. (2002), who employed a statistical model to discover the genetic origin of the allometric relationship between stem height and stem biomass in F_2_ populations of poplar. Utilising a dynamic allometric QTL mapping model, Li et al. (2007) further analysed the genetic mechanism of ontogenetic C allocation in soybean RIL populations. Based on the statistical and/or dynamic models, the QTLs related to biomass distribution and balanced growth between plant organs or traits were subsequently identified for the aboveground-underground growth of *Populus euphratica* (Zhang et al., 2017), leaf number and whole dry weight of *Arabidopsis* (Gan et al., 2019) as well as for the leaf area-leaf dry weight of common bean (Zhang et al., 2020b). Despite these advances, genetic analyses of C allocation have focused mainly on annual herbs and crops. In addition, most of the studies considered relatively few traits, while plant traits are multiple and complex, generally interacting with each other. It is therefore necessary to identify QTLs in order to elucidate C allocation patterns by considering the phenotypical diversity of woody plants.

*Catalpa bungei* C. A. Mey. is a precious timber and garden ornamental tree species that is widely distributed in the temperate, subtropical and tropical regions of China (Wang et al., 2016). This tree species has considerable genotype variation with regard to growth performance over the long-term evolution process (Wang et al., 2016; Lu et al., 2019). To identify major covariation QTLs for biomass allocation between leaf and stem growth of *C. bungei*, we constructed a bivariate mapping model to detect covariation QTLs that govern trait covariance via C allocation among and within plant organs in a full-sib mapping population of this tree species. Candidate gene analysis of significant QTLs was further conducted, utilising tissue-specific gene expression data. The discovery of covariation QTLs related to biomass allocation between leaves and stems will help elucidate the genetic mechanism of plant morphogenesis and will provide a theoretical basis for forest molecular marker-assisted breeding with specific C allocation strategies depending on the heterogeneous environments.

## Materials and Methods

### Mapping Population and SNP Genotyping

A full-sib population of 200 lines was generated from the cross between *C. bungei* “7080” (female parent) and *C. duclouxii* “16-PJ-3” (male parent). The parents and progenies were propagated via bud grafting and then planted in the experimental field in Luoyang, China (N 112.55°, E 34.71°) in 2018, following a randomised block design. For all 200 lines, a genome-wide panel of single nucleotide polymorphisms (SNPs) was sequenced through restriction-site associated DNA (RAD) technology using an Illumina HiSeq X Ten platform. The genetic map was constructed using a set of 9,593 SNPs, following several strict criteria in our previous study. The integrated genetic map included 20 linkage groups and spanned 3,151.63 cM, with an average distance of 0.32 cM between adjacent markers (Lu et al., 2019). After further filtering for duplicate markers due to imputation missing values, 6,446 SNPs were retained for this final study, including 5,362 testcross and 1,084 intercross type markers, referring to the segregation ratios of 1:1 and 1:2:1. Genotyping data were submitted to the NCBI SRA database (http://www.ncbi.nlm.nih.gov/sra) under accession number PRJNA551333.

### Phenotyping

A randomised block design was applied to the F_1_ population, with two ramets per clone in each plot and five replicates. Leaf traits of the third whorl of fully expanded leaves were evaluated on 2018/9/5 at the end of the rapid growth period. Six leaf traits were assessed, namely leaf length (LL), leaf width (LW), leaf length/width ratio (L/W), leaf area (LA), leaf perimeter (LP) and petiole length (PL). Stem growth traits were observed on 2018/10/10 sat the end of the growing season and included tree height (H) and diameter at breast height (DBH). The measurement methods for these phenotypic data were previously described in detail by Lu et al. (2019).

Wood density was measured during October 2018 after harvest. Stem segments from at least three individuals of each line were excised at 125–130 cm above ground level. After removing the bark and pith with a razor blade, wood density was determined from volumetric displacement and oven-dried mass at 103 ± 3°C for at least 6 h (Cornelissen et al., 2003). The ratio of dry weight to volume is the wood density (WD, g/cm^3^). Stemwood volume (V) in forest trees is an important trait for forest productivity. This trait is determined by stem height (H) and stem diameter at breast height (DBH) through a geometric function, expressed as the below equation, which is modified from Sang et al. (2019):

$$V=0.785\times {\left(DBH\right)}^{2}\times H$$

The stemwood biomass (SB) can be calculated by SB=V×BD .

### QTL Analysis via Bivariate Mapping

#### QTL Mapping

The constructed linkage map is quite dense, and we employed a multiplicative model that assumes QTLs are located at the positions of markers. The sample size of the mapping population is *n*, and the phenotypic values of individual *i* are *y*_*i*_. Two phenotypic variables were selected and combined within or between leaf and stem traits. The multiplicative likelihood model is expressed as:

$$L\left(\Phi |y\right)\prod_{i=1}^{n_{1}}f_{1}\left(y_{i}\right)\ldots \prod_{i=1}^{n_{j}}f_{j}\left(y_{i}\right)$$ (1)

where Φ is the unknown parameter, **y**_*i*_ = (*y*_1*i*_, *y*_2*i*_) is the phenotypic vector of progeny *i* for trait 1 (coded by 1) and trait 2 (coded by 2), *n*_*j*_ is the number of progeny with SNP genotype *j*, and *f*_*j*_(y_*i*_) is a bivariate normal distribution for progeny *i* with the expected mean vector for genotype *j* (*µ*_1*j*_, *µ*_2*j*_) and the variance-covariance matrix Σ .

$$\text{Σ}=\left(\begin{array}{cc} \sigma_{1}^{2} & \rho \sigma_{1}\sigma_{2}\\ \rho \sigma_{1}\sigma_{2} & \sigma_{2}^{2} \end{array}\right)$$ (2)

where $\sigma_{1}^{2}$ and $\sigma_{2}^{2}$ are the variances of two different traits, and ρ is the trait-trait correlation. Statistical methods based on likelihood (1) have been established to estimate the model parameters Φ = (*µ*_1*j*_, *µ*_2*j*_, $\sigma_{1}^{2}$ , $\sigma_{2}^{2}$ , ρ ).

#### Hypothesis Tests

After scanning SNP loci on the linkage map, we can detect whether a significant *cov*QTL affects the C allocation variation of leaf traits and stem traits as well as the covariation between leaves and stems. The tests are based on the following hypotheses:

$$\begin{array}{l} H_{\text{0}}\text{:}\left({µ}_{1j}{\text{, }µ}_{2j}\right)=\left({µ}_{1}{\text{, }µ}_{2}\right)\text{, for j }=\;1\text{ or }2\\ H_{1}\text{: At least one equality above does not hold} \end{array}$$ (3)

The log-likelihood ratio (LR) under H_0_ (there is no QTL) and H_1_ (there is a QTL) will be calculated. Significant QTLs were identified by comparing LR values with a critical threshold. The threshold can be empirically determined from permutation tests by reshuffling the phenotypic data 1,000 times (Churchill and Doerge, 1994). The top 5% of the maximum LR values were used as a critical threshold to indicate a significance level of 0.05.

Exploratory data analysis and visualisation, including curve fitting, correlation analysis, QTL mapping and epistasis detection, were performed using R-project version 4.0.3 (R Core Team, 2018), ggplot2 (3.3.2) and dplyr (1.0.2). Possible functions for all QTLs determined were annotated and predicted via BLAST in the “nr” database on the National Center of Biotechnology Information website (NCBI; http://blast.ncbi.nlm.nih.gov/), identified on Uniprot (http://www.uniprot.org/) and analysed for protein-protein interactions using online protein interactions analysis software STRING (http://string-db.org/).

### Expression Analysis of Candidate Genes

Transcriptomic characterisation of biomass allocation *cov*QTLs identifies candidate genes that regulate the growth and development of specific organs in *C. bungei*. Different tissues (leaf, petiole, xylem and phloem) were collected from a more than one-hundred-year-old *C. bungei* in Nanyang, Henan province. RNA extraction was performed using the Plant Qiagen RNeasy kit according to the manufacturer’s instructions. RNA sequencing libraries were constructed using an Illumina standard mRNA-Seq Prep Kit, and sequence data were obtained using the Illumina HiSeq 2500 platform (Illumina Inc., San Diego, CA, United States) to obtain 2 × 125 bp reads. Raw data were trimmed to remove adaptors, and reads of less than 100 bp were discarded to enhance the quality. The clean reads were mapped to the *C. bungei* genome using the TopHat2 package with default parameters and assembled using Cufflinks. Gene expression was expressed as fragments per kilobase of transcript per million fragments mapped reads (FPKM) values. To verify the significant QTLs, FPKM values for each candidate gene were computed to assess expression across four tissues, and the FPKM values were transformed using a log2 fold change. Fold changes in the expression of candidate genes were used to generate a gene expression heatmap using the R package “pheatmap”.

## Results

### How Trait-Trait Relationships Vary

**FIGURE 1:**
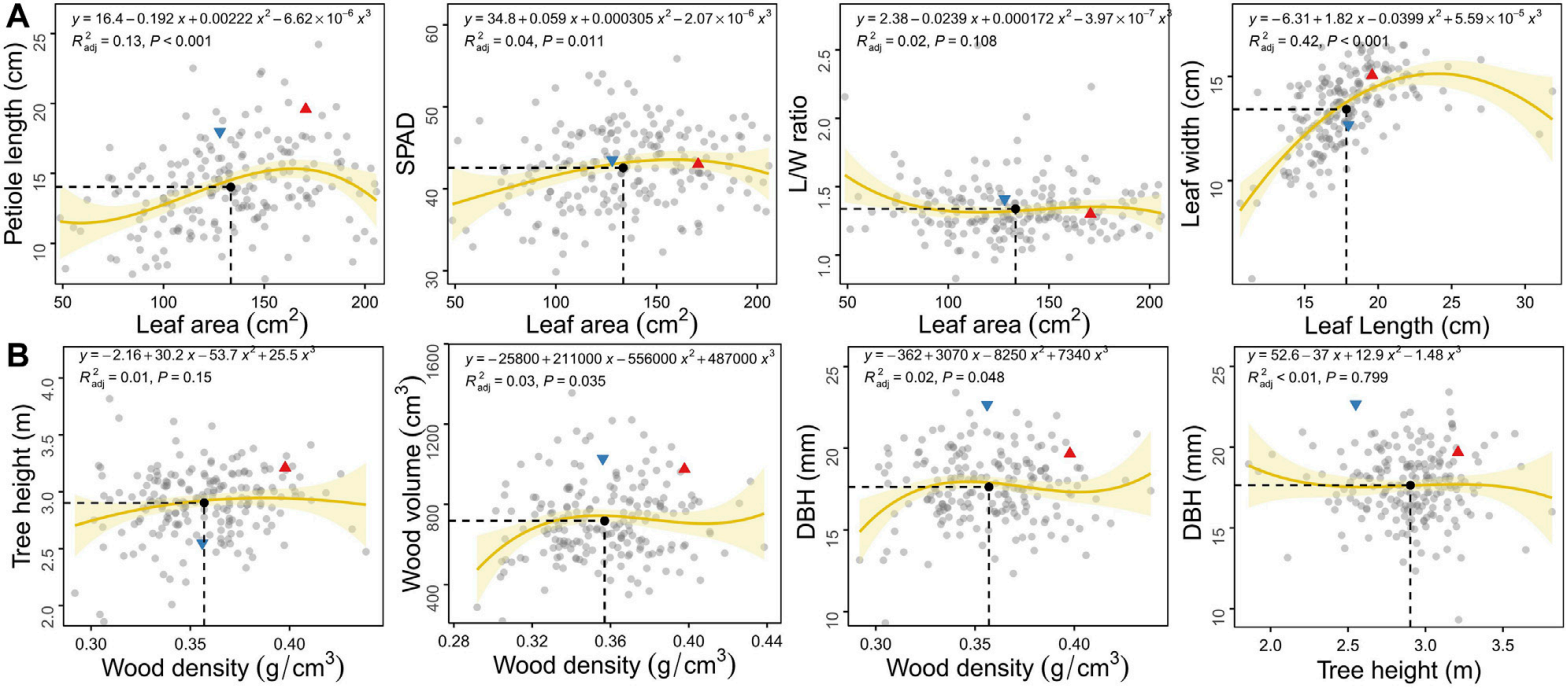
Scatter plots of growth traits within organs of the leaf **(A)** and stem **(B)** in the F_1_ population of *C. bungei*. The red triangle and blue inverted triangle denote the original parent plants “7080” and “16-PJ-3”, respectively. The mean value of each trait pair among all individuals is indicated by the black solid circle crossed by vertical slash line and horizontal slash line.

Through plotting the correlation between traits within leaves and stem and fitting the curve by polynomial regression, we can visualise how biomass allocation influences the morphology and function of organs. In general, all growth traits exhibited considerable genotypic variation among the F_1_ population. The great genetic variation was observed in the leaf area compared to the other three leaf traits (Figure 1A) as well as in wood density compared to the other three stem traits (Figure 1B). The male parent “16-PJ-3” showed a smaller leaf size and wood base density than the female parent “7080”. There were little differences in leaf function (SPAD), leaf morphology (L/W ratio), wood volume and stem mass between parents. Neither accession was completely distributed on the two sides on the diagonal of mean values. For each trait-trait pair, numerous transgressive segregants beyond the range of two original parents were detected, indicating that some alleles increased the phenotypic values and others decreased the values.

Relationships between leaf area and other traits (petiole length, SPAD and L/W ratio) indicated the balanced growth of leaf biomass allocation, photosynthetic function and leaf morphology, respectively. At the same mean value of leaf area (13.34 cm^2^), leaves tended to maintain higher photosynthetic function instead of petiole elongation and leaf morphology (L/W ratio, Figure 1A). According to the fitting curve, petioles fluctuated with the increase in leaf area, gradually increased to the plateau stage and then began to decrease. Some individuals had large leaf areas (>180 cm^2^) and relatively short petioles (<13 cm). The SPAD and L/W ratios changed little with leaf area, the SPAD content of small leaves was lower, and the leaf shape was longer and narrower. In terms of biomass allocation between leaf length and width, greater blade width was observed as the length increased at the beginning. However, leaf width decreased when LL > 24 cm.

By analysing the relationship between wood density and primary growth (H), wood volume (V) as well as secondary growth (DBH), the C allocation tradeoff between stem cell number and volume was demonstrated. Tree radial growth increased slightly, gradually plateaued and finally declined slightly (Figure 1B). Similar to WD-DBH and WD-V, thinner and smaller trees also had lower wood density. The C allocation between plant height and DBH was very important for plant morphology. For the current population, the variation between tree height and DBH generally decreased with greater plant height. Biomass was preferentially allocated to wood density, rather than tree height.

**FIGURE 2:**
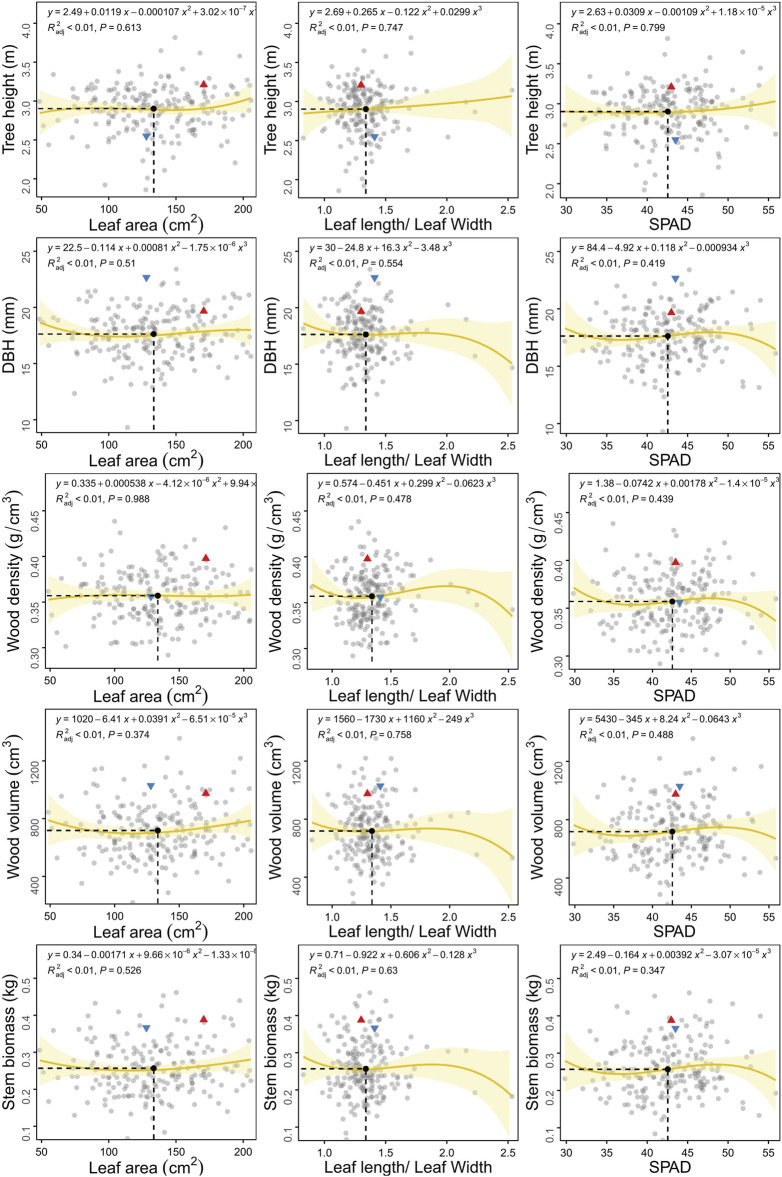
Scatter plots of growth traits between leaf and stem in the F_1_ population of *C. bungei*. The red triangle and blue inverted triangle denote the original parent plants “7080” and “16-PJ-3”, respectively. The mean value of each trait pair among all individuals is indicated by the black solid circle crossed by vertical slash line and horizontal slash line.

Biomass partitioning between leaves and stems could reflect the connection between source and sink as well as the functional tradeoff between sunlight interception by tree height and photosynthesis by leaves (Figure 2). We selected three leaf traits, LA, L/W and SPAD, which could represent the three aspects of leaf biomass, morphology and function, respectively. We then performed correlation analysis between leaf and stem traits. Stem elongation, thickening and wood density did not change with an increase in the area of a single leaf. This phenomenon demonstrates that the leaf area of the whole tree should be considered rather than that of a single leaf. Tree height increased with leaf area, leaf morphology and SPAD, indicating that larger, longer and higher SPAD leaves could promote faster axial growth. The influence of leaf morphology (L/W) on stem radial growth, biomass, wood density and volume was more dramatic than growth in height. The leaves of individuals with greater DBH were shallow rectangular (L/W < 1), while the leaves of those with a lower DBH were more elongated (L/W > 2.2). The relationship between leaf photosynthesis and stem phenotype varied in a manner similar to that for leaf morphology, yet with less fluctuation. Individuals with slower radial growth and lower wood density had more SPAD in the leaves in order to compete for light resources and enhance photosynthesis.

### How *cov*QTLs Govern Trait-Trait Covariance

**FIGURE 3:**
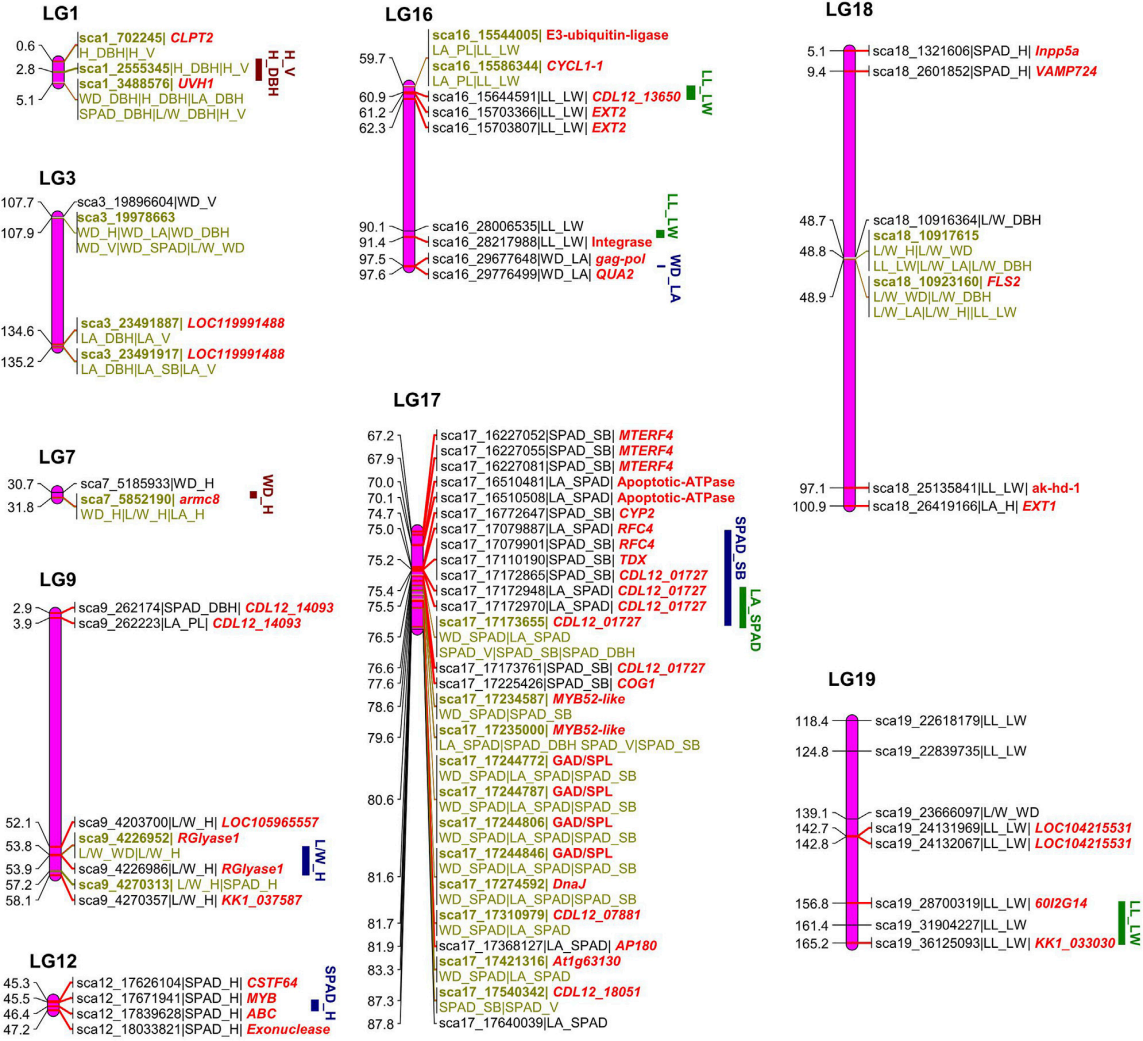
Diagrammatic genomic positions of significant covariation quantitative trait loci (*cov*QTLs) detected for trait-trait pairs of leaf and stem growth.

The bivariate mapping model identified 123 significant *cov*QTLs for all trait pairs, including 67 testcross and 57 intercross type SNPs (Supplementary Figure S1, Supplementary Table S1), of which 73 unique SNPs remained after removing duplications. A total of 38 *cov*QTLs (24 testcross and 13 intercross type) were identified for trait-trait pairs in the leaf. There were 3, 16, 2 and 17 QTLs for LA-PL, LA-SPAD, L/W-LA and LL-LW, respectively, which were mainly located on lg16-lg19. The *cov*QTLs of C allocation for LL-LW were located at intervals of 59.7–91.4 cM on lg16 and 118.4–165.2 cM on lg19 (Figure 3). In addition, the QTLs of sca18_10917615 and sca18_10923160 were co-located with L/W-LA, indicating the reliability of mapping results based on similar traits. Coordinated variation between leaf area and leaf function (LA-SPAD) was observed relative to the interval of 70.0–87.8 cM on lg17 with peak QTL sca17_17310979 at 81.7 cM, overlapping with the stem biomass and leaf function (SB-SPAD). Regarding *cov*QTL mapping for the stem, 11 intercross type SNPs were discovered on lg1, 3, 7 and 15 for the trait pairs of H-DBH, WD-DBH, WD-V and WD-H. The C allocation of H-DBH was regulated by the QTL region of 0.6–5.1 cM on lg1 with the peak QTL sca1_3488576. Bivariate mapping was used to analyse significant QTLs for covariance between leaves and stem, resulting in a total of 71 significant *cov*QTLs (46 unique SNPs after removing duplications) related to 13 trait-trait pairs (Supplementary Table S1). Several significant QTL regions were detected in lg9, 12 and 17 for L/W-H, SPAD-H, and SPAD-SM. There were no QTLs for L/W-SB or L/W-V.

The functional annotations of 73 significant *cov*QTLs were identified via BLAST in the “nr” database on NCBI, with 44 gene terms after removing duplications for all trait pairs (Figure 3). The putative genes were mainly aligned to the genomes of *Sesamum indicum*, *Populus deltoids*, *Handroanthus impetiginosus* and *Erythranthe guttata* (Supplementary Table S1). Several important genes were annotated by multiple QTLs (Supplementary Table S1; Figure 3). QTLs sca9_4226952 and sca9_4226986 related to L/W-H and SPAD-H, respectively, encode rhamnogalacturonate lyase B (*rglB*), which is involved in the carbohydrate metabolic process and catalytic activity. Three QTLs related to SPAD-SM in a narrow QTL region of 67.2–70.0 cM on lg17 encode the mitochondrial transcription termination factor 4 (MTERF4), which participates in the regulation of mitotic cell cycle spindle assembly checkpoint. Four QTLs between 80.6–81.6 were related to glutamate decarboxylase/sphingosine phosphate lyase (*GAD*/*SPL*), playing a key role in the carboxylic acid metabolic process. The replication factor C subunit, *RFC4*, which was related to sca17_17079887 and sca17_17079901, participates in the biological process of DNA biosynthesis, DNA replication, ATP binding and nucleotidyltransferase activity as part of the DNA polymerase III complex. More importantly, some *cov*QTLs were co-located for several trait-trait pairs, which were labelled on the linkage map (Figure 3). Among them, sca1_3488576, sca3_19978663, sca3_23491917, sca17_17173655, sca17_17235000, sca18_10917615 and sca18_10923160 were associated with more than three trait pairs, suggesting that they may play an important role in C allocation and coordinated growth.

### How Biomass Allocation-Related covQTLs Remould the Tree Growth Model

**FIGURE 4:**
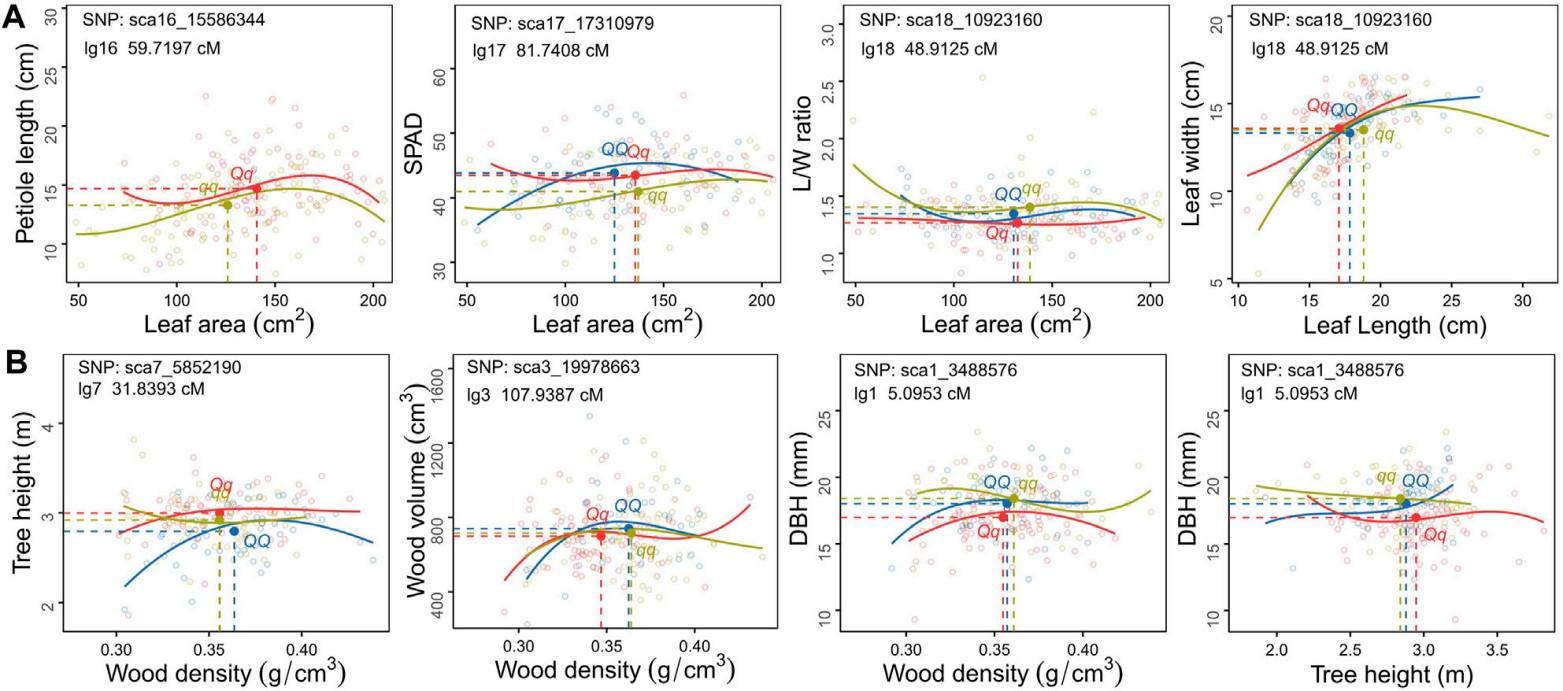
Scatter plots of carbon allocation between traits within leaf **(A)** and stem **(B)** organs for different *C. bungei* genotypes. The mean values of each trait pair of different genotype populations is indicated by the blue (*QQ*), red (*Qq*) and ginger (*qq*) solid circles crossed by vertical slash lines and horizontal slash lines.

**FIGURE 5:**
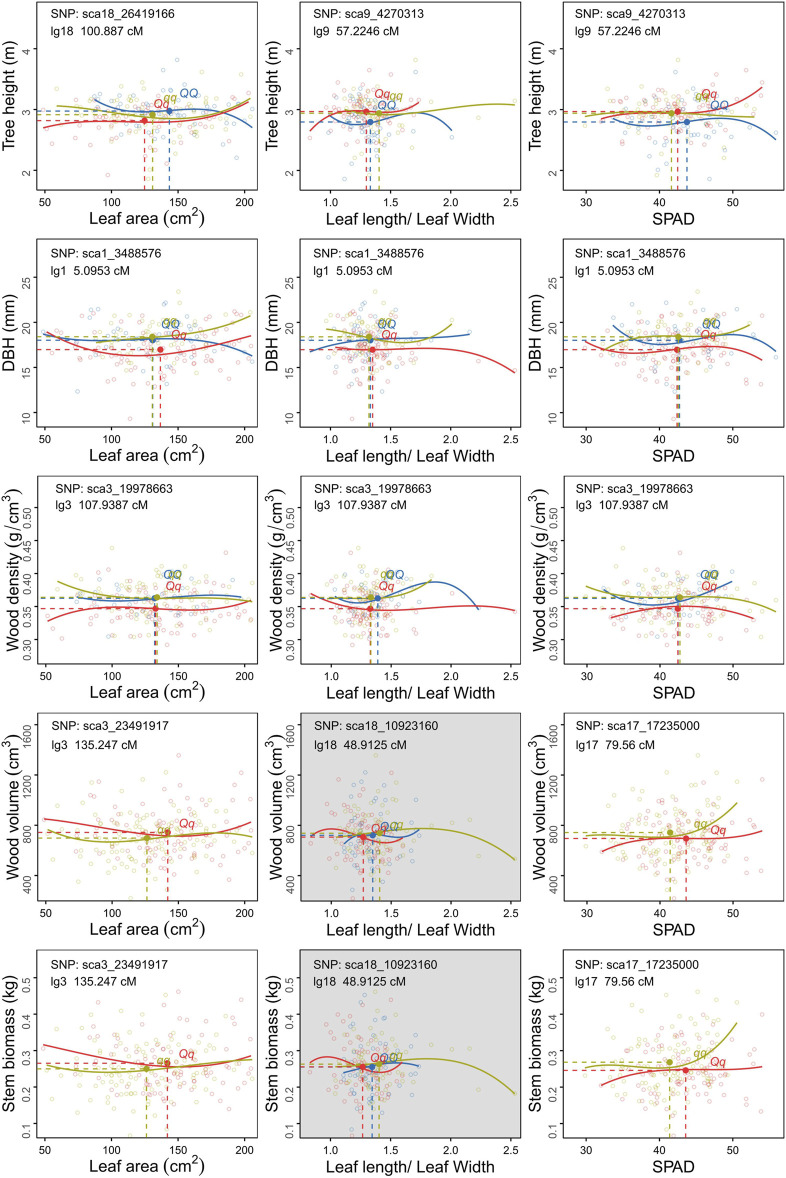
Scatter plots of carbon allocation between leaf and stem traits of different genotype populations of *C. bungei*. The mean values of each trait pair of different genotypes populations is indicated by the blue (*QQ*), red (*Qq*) and ginger (*qq*) solid circles crossed by vertical slash lines and horizontal slash lines.

Genotype-specific trait-trait correlation analyses were conducted for each *cov*QTL with a maximum LR value (Figures 4, 5). Among 200 accessions, two or three groups could be established based on the testcross or intercross type. The cyclin-L1-1 (*CYCL1-1*) gene, located within sca16_15586344, participates in cell division, transcription regulation by RNA polymerase II and catalytic activity. Individuals with genotype *Qq* exhibited larger leaf size and longer petioles than those with genotype *qq*. For *cov*QTL sca17_17310979, the *Qq* genotype had a higher SPAD than *qq* at a similar leaf area, while having a larger leaf area than *QQ* at approximately equal SPAD, suggestive of allelic additive effects for the trade-off between leaf area and SAPD. The marker sca18_10923160 was co-located for five trait pairs, which was the peak QTL for L/W-LA and LL-LW (Figure 1A). LRR receptor-like serine/threonine-protein kinase *FLS2*, detected in four trait pairs, including L/W-WD, L/W-DBH, L/W-H and L/W-LA, was associated with the defense response via callose deposition in the cell wall, receptor-mediated endocytosis and anion channel activity (Table 1; Supplementary Tables S2, S3).

**TABLE 1:** The positions, LR values and functional annotation are shown for the significant SNPs that affect biomass allocation within or between leaf and stem growth of *C. bungei*.

No	Trait pairs	SNP	LG	Position (cM)	LR	Annotation
1	WD-DBH	sca1_3488576	1	5.10	19.39	*UVH1*
2	WD-H	sca7_5852190	7	31.84	18.82	*armc8*
3	WD-LA	sca16_29677648	16	97.54	16.12	*gag-pol*
4	WD-LA	sca16_29776499	16	97.62	17.38	*QUA2*
5	WD-SPAD	sca17_17234587	17	78.56	15.80	*MYB52-like*
7	WD-SPAD	sca17_17244787	17	80.56	16.58	*GAD/SPL*
10	WD-SPAD	sca17_17274592	17	81.61	16.43	*DnaJ*
12	WD-V	sca15_9179490	15	39.33	16.76	*CFOL_v3_36453*
13	H-DBH	sca1_702245	1	0.64	18.54	*CLPT2*
14	H-DBH	sca1_3488576	1	5.10	22.47	*UVH1*
15	H-V	sca1_702245	1	0.64	18.23	*CLPT2*
16	H-V	sca1_3488576	1	5.10	21.61	*UVH1*
17	L/W-WD	sca9_4226952	9	53.81	18.72	*rglB*
18	L/W-WD	sca18_10923160	18	48.91	18.51	*FLS2*
19	L/W-DBH	sca18_10923160	18	48.91	18.57	*FLS2*
20	L/W-H	sca7_5852190	7	31.84	16.94	*armc8*
21	L/W-H	sca9_4226952	9	53.81	20.95	*rglB*
25	L/W-H	sca18_10923160	18	48.91	17.45	*FLS2*
26	L/W-LA	sca18_10923160	18	48.91	18.03	*FLS2*
27	LA-DBH	sca1_3488576	1	5.10	21.40	*UVH1*
28	LA-H	sca7_5852190	7	31.84	18.10	*armc8*
29	LA-H	sca18_26419166	18	100.89	18.25	*EXT1*
30	LA-PL	sca16_15544005	16	59.65	17.24	*E3 ubiquitin ligase*
31	LA-PL	sca16_15586344	16	59.72	17.27	*CYCL1-1*
33	LA-SPAD	sca17_16510508	17	70.08	18.29	*Apoptotic ATPase*
34	LA-SPAD	sca17_17079887	17	75.05	17.68	*RFC4*
35	LA-SPAD	sca17_17235000	17	79.56	16.50	*MYB52-like*
37	LA-SPAD	sca17_17244787	17	80.56	18.97	*GAD/SPL*
40	LA-SPAD	sca17_17274592	17	81.61	18.62	*DnaJ*
41	LA-SPAD	sca17_17368127	17	81.93	16.88	*AP180*
43	LL-LW	sca16_15544005	16	59.65	14.90	*E3 ubiquitin ligase*
44	LL-LW	sca16_15586344	16	59.72	15.72	*CYCL1-1*
46	LL-LW	sca16_15703807	16	62.35	14.67	*EXT2*
47	LL-LW	sca16_28217988	16	91.45	16.75	*Integrase*
48	LL-LW	sca18_10923160	18	48.91	18.04	*FLS2*
49	LL-LW	sca18_25135841	18	97.10	16.74	*ak-hd 1*
50	LL-LW	sca19_28700319	19	156.76	14.02	*60I2G14*
51	SPAD-DBH	sca1_3488576	1	5.10	18.43	*UVH1*
52	SPAD-DBH	sca17_17235000	17	79.56	14.34	*MYB52-like*
53	SPAD-H	sca12_17626104	12	45.27	16.72	*CSTF64*
54	SPAD-H	sca12_17671941	12	45.49	16.23	*MYB*
55	SPAD-H	sca12_17839628	12	46.39	16.48	*ABC*
56	SPAD-H	sca12_18033821	12	47.22	17.05	*Exonuclease*
57	SPAD-H	sca18_1321606	18	5.07	16.56	*Inpp5a*
58	SPAD-H	sca18_2601852	18	9.39	17.18	*VAMP724*
60	SPAD-SB	sca17_16227055	17	67.89	17.21	*MTERF4*
62	SPAD-SB	sca17_16772647	17	74.69	17.25	*CYP2*
63	SPAD-SB	sca17_17079901	17	75.19	16.80	*RFC4*
64	SPAD-SB	sca17_17110190	17	75.21	16.89	*TDX*
65	SPAD-SB	sca17_17225426	17	77.56	16.95	*COG1*
67	SPAD-SB	sca17_17235000	17	79.56	18.43	*MYB52-like*
68	SPAD-SB	sca17_17244772	17	80.56	18.05	*GAD/SPL*
72	SPAD-SB	sca17_17274592	17	81.61	17.34	*DnaJ*
73	SPAD-V	sca17_17235000	17	79.56	16.07	*MYB52-like*

As for the covariation between leaf and stem, the degrees of variation among genotypes *QQ*, *Qq* and *qq* of sca1_3488576 were distinct among LL-DBH, L/W- DBH and SPAD-DBH (Figure 5). The marker sca17_17235000 encodes transcription factor MYB52-like, which had the maximum LR value in SPAD-V and SPAD-SB. The mean values and fitting curves of different genotypes were similar for SPAD-V and SPAD-SB at sca17_17235000 and sca3_23491917. For the trade-off between tree height and leaf traits, the co-located marker sca9_4270313 displayed different patterns among genotypes, as genotype *Qq* with the smallest L/W and medium SPAD tended to promote plant height (Figure 5). The most significant *cov*QTL sca3_19978663 was co-located with three pairs of WD-leaf traits (LA, L/W and SPAD). Populations with genotypes *QQ* and *qq* displayed higher wood density and DBH than *Qq* at similar leaf areas and SPAD.

The *cov*QTL sca1_3488576 with the maximum LR value was co-located for five trait pairs, including WD_DBH, H-DBH, LA-DBH, SPAD-DBH and L/W-DBH (Figures 4B, 5). This marker was related to the gene encoding DNA repair endonuclease UVH1 isoform X1 (*UVH1*), which is involved in nucleic acid phosphodiester bond hydrolysis and endonuclease activity. For the C distribution of WD-DBH and H-DBH, *UVH1* (sca1_3488576) plays different regulatory roles. At the same DBH value, the subpopulation of genotype *qq* tended to invest more photoassimilates to wood density than tree height, while genotype *Qq* preferentially allocated photoassimilates to tree height instead of wood density (Figure 4B) and also allocated more nutrition for leaf area expansion instead of SAPD increase (Figure 5).

### Expression of Candidate Genes Associated With Trait-Trait Relationships

**FIGURE 6:**
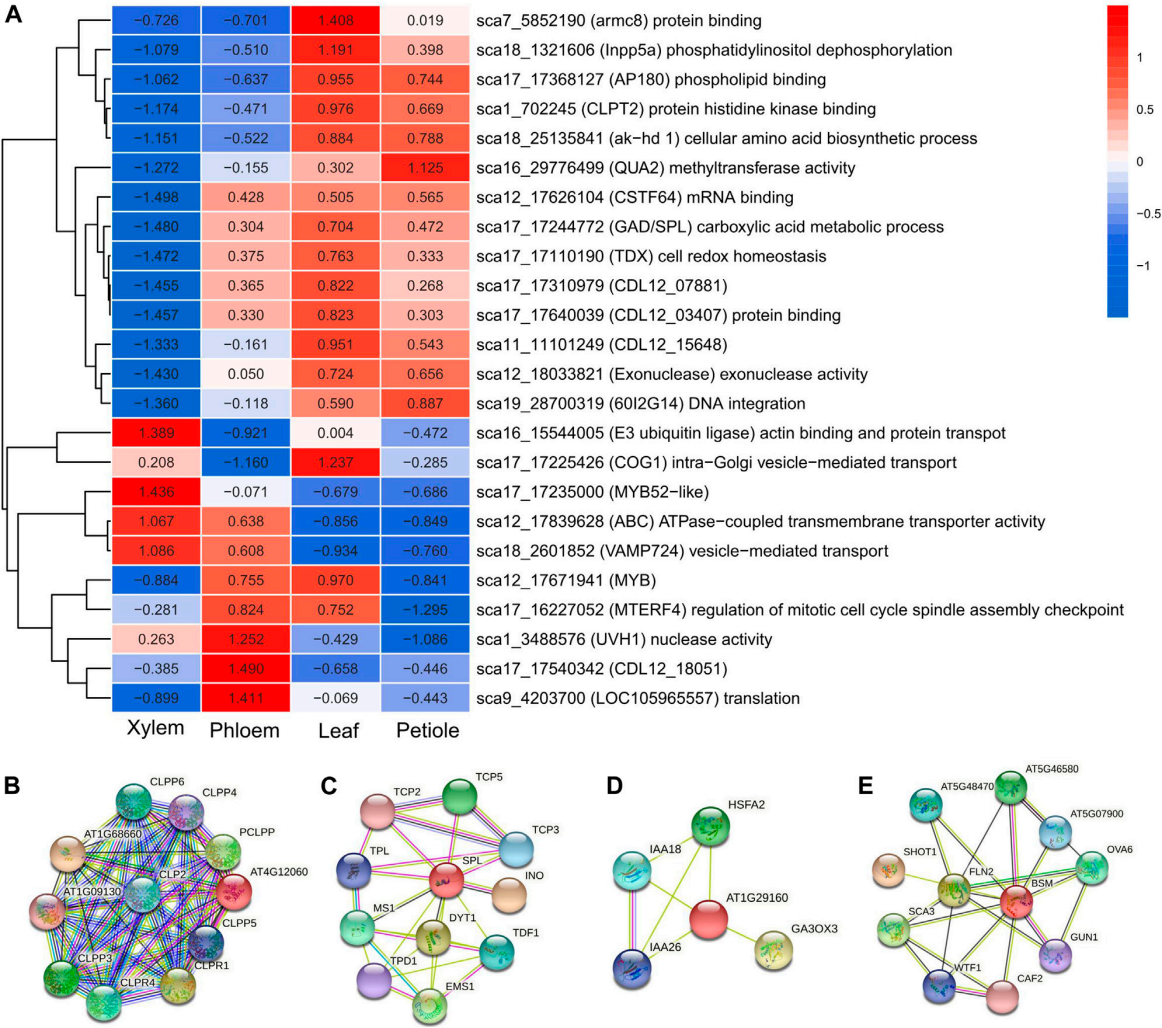
**(A)** Heat map of the gene expression analysis for 24 biomass distribution-related genes using RNA-seq data from four tissues of *C. bungei.* **(B–E)** Interaction network of *CLPT2*, *SPL*, *COG1* (*At1g29160*) and *MTERF4* (*BSM*) in *Arabidopsis thaliana*.

We investigated the expression profiles of the 123 *cov*QTLs to assess the overlapping genes via QTL and transcriptomic approaches. A total of 24 candidate genes were found to be significant in all trait-trait pairs (Figure 6A). Cluster analysis of gene expression values revealed two gene clusters. Most of the Cluster I genes were upregulated in the leaf tissues and downregulated in stem tissues, with related candidate *cov*QTLs also mainly detected in leaf trait pairs, including sca17_17368127, sca17_17640039 and sca17_17244772 for LA-SPAD, sca18_25135841 and sca19_28700319 for LL-LW. These genes are involved in the biological processes of biomacromolecule synthesis, such as phospholipid binding and endocytosis (*AP180* and *Inpp5a*), protein binding (*CDL12_03407* and *armc8*), cellular amino acid biosynthetic process (*ak*−*hd1*), DNA integration (6*0I2G14*), exonuclease activity (Exonuclease) and methyltransferase activity (*QUA2*).

ATP-dependent Clp protease ATP-binding subunit *CLPT2* (sca1_702245) is an important component of the chloroplast, acting as an accessory protein that regulates the assembly of the plastidial Clp protease system, which is involved in the protein interaction network of proteolysis protein metabolic process and chloroplast organisation. *CLPT2* (*At4g12060*) was upregulated only in leaves (Figure 6B; Supplementary Tables S2, S3). As a subunit of the Clp core complex, *CLPT2* plays an important role in leaf growth and development, including leaf colour, shape and growth rate (Kim et al., 2015). Several genes with high expression levels in both leaves (leaf and petiole) and stem (phloem), such as *CSTF64* (sca12_17626104), *GAD*/*SPL* (sca17_17244772) and *TDX* (sca17_17110190), were involved in the processes of mRNA binding, carboxylic acid metabolism and cell redox homeostasis, respectively. *GAD*/*SPL* was the key gene related to several trait pairs and covers, being involved in floral organ development, floral organ morphogenesis and formation, regulation of shoot apical meristem development as well as macromolecule metabolic processes such as nitrogen compound and RNA metabolism. *SPL*, *YAB*3 and *AFO* likely have transcription factor activity and were involved in the abaxial cell fate determination during embryogenesis and organogenesis (Figure 6C; Supplementary Tables S2, S3).

All Cluster II genes were downregulated in the petiole and half in the leaves. In contrast, most of these genes were upregulated in the phloem and half in the xylem. We observed that sca16_15544005 (*E3 ubiquitin ligase*), sca17_17225426 (*COG1*), sca12_17839628 (*ABC*) and sca18_2601852 (*VAMP724*), which are implicated involved in substance transportation, were highly expressed in the xylem. E3 ubiquitin ligase enables actin binding and ligase activity, thus being involved in protein transport. ABC participates in ATPase-coupled transmembrane transporter activity and ATP binding. Vesicle-associated membrane protein 724 (*VAMP724*) is involved in vesicle-mediated transport. *COG1* is upregulated in both leaves and xylem, consistent with the related trait pair of SPAD-stem biomass.

*COG1* is involved in intra-Golgi vesicle-mediated transport as part of the Golgi transport complex located on the membrane of the Golgi apparatus. STRING analysis of *COG1* (*At1g29160*) indicated that it acts as a negative regulator of phytochrome-mediated light responses. The interactive protein *GA3OX3* is involved in the production of bioactive GA by converting inactive gibberellin precursors *GA9* and *GA20* in the bioactive gibberellins GA4 and GA1. *PHYA* and *PHYB* are regulatory photoreceptors that exist in two forms, Pr and Pfr, which are reversibly interconvertible by light. Photoconversion of Pr to Pfr induces an array of morphogenetic responses, whereas the reconversion of Pfr to Pr cancels the induction of these responses (Figure 6D; Supplementary Tables S2, S3). *MTERF4* (sca18_2601852) regulates mitotic cell cycle spindle assembly checkpoint and chloroplast organisation, showing high expression in the leaves and phloem at the same time. *MTERF4* (*BSM*) is required for the maturation of 16S rRNA and 23S rRNA in the chloroplast, thus maintaining appropriate transcript levels in the mitochondria and chloroplasts. *MTERF4* is involved in the regulation of cellular macromolecule biosynthetic process, nitrogen compound metabolic process, primary metabolic process, transcription and gene expression. Interactive proteins *FLN1* and *FLN2* of *BSM* were required for proper chloroplast development (Figure 6E; Supplementary Tables S2, S3).

## Discussion

The coordinated growth and biomass allocation among organs determine their adaptability to the environment, which is of major significance to the economic, ecological and social aspects of plant cultivation (Ambrose et al., 2009; Zhang and Xi, 2021). Plants maximise overall growth through the optimal utilisation and allocation of resources available within their environment, thus leading to the growth of different organs at distinct rates (Poorter et al., 2012). Based on the F_1_ population of the woody plant *C. bungei*, different trade-off patterns were observed for various trait pairs (Figures 1, 2). We evaluated leaf phenotypic data for a single leaf, which is not representative of the total leaf biomass for the whole plant. Therefore, there was no significant linear relationship between leaf and stem phenotypes. We used polynomial regression curve fitting to infer potential relationships between traits. In most trait combination pairs, the parents were clearly separated among the population, except for leaf L/W ratio, wood volume, stem biomass and SPAD. At the mean value of leaf area, *C. bungei* prioritised C allocation to increase SPAD, that is, chlorophyll content, which directly improved photosynthesis (Niinemets, 2007). Petiole elongation is the second preferred method for ensuring the appropriate spatial distribution of leaves to intercept more light energy (Levionnois et al., 2020). This C distribution strategy indicated that the plant accelerated vegetative and biomass accumulation at the sapling/early stage through leaf area expansion to increase photosynthetic efficiency (Liu et al., 2020).

Organisms tend to increase their surface area to enhance metabolic capacity in an attempt to produce sufficient matter and energy for survival and reproduction (Wang and Wu, 2004). At the same mean value of DBH (17.63 mm), individuals preferentially invested more nutrients into height (2.90 m) for increasing the surface area, rather than in wood density (0.36 g/cm^3^). This allocation pattern is consistent with the growth law of woody plants (Spicer and Groover, 2010). The trees prioritise primary growth at the early juvenile stage, which refers to the elongation growth of plant organs caused by the division, differentiation and growth of the apical meristem cells of roots and stems (Zhang et al., 2017). After the completion of primary growth, most dicotyledonous plants accelerate the secondary growth process of continuously producing secondary xylem and secondary phloem with cambium activity, resulting in a thickened root and stem diameter as well as greater tissue density (Spicer and Groover, 2010). As per leaf growth and development, trees in the present study tended to develop longer leaves, with mean values of leaf length and width at 17.84 and 13.43 cm, respectively. The relationship between leaf morphology traits requires further study in different populations or growth stages.

Previous studies on genetic variation in relation to the growth traits and genotype-by-environment of *C. bungei* indicated that the height and stem volume of *C. bungei* were under strong genetic control (Xiao et al., 2019). The genetic basis of coordinated growth and trade-off strategies among growth traits have since attracted great interest among biologists. However, the conventional QTL mapping method focusing on genetic variation at a single trait may fail to reveal the genetic architecture underlying trait-trait balanced growth (Gan et al., 2019; Zhang et al., 2020b). Currently, there are two statistical approaches for QTL mapping, namely the mixture model for sparse molecular markers and the multiplicative model for dense markers. Inclusive composite interval mapping with a mixture model was carried out to detect QTL regions related to a single growth trait in a previous study (Lu et al., 2019). Because the constructed linkage map was quite dense, we employed bivariate mapping with the multiplicative model that assumes pleiotropic QTLs were located at the positions of markers. Compared with single-trait QTL mapping, *cov*QTL analysis considers two-dimensional phenotypic data at the same time to address the complexity and multiplicity of the trait, thus improving the accuracy and efficiency of QTL mapping. We detected 123 *cov*QTLs for all trait-trait pairs, some of which were consistent with previous QTL mapping results. The *cov*QTL of C allocation trade-off of leaf traits on lg16, 17, 18 and 19 overlapped with the intervals of Q16-60, Q17-84, Q18-99 and Q19-137 reported for leaf traits in a previous study (Lu et al., 2019). Further, we also detected some important *cov*QTLs which influenced the C distribution between stem traits or stem-leaf traits, such as the QTL region of 0.6–5.1 cM on lg1 for H-DBH as well as regions on lg9, 12 and 17 for L/W-H, SPAD-H and SPAD-SM, respectively. It should be noted that the region with density SNPs between 76.5 and 87.8 CM on lg17 was associated with multiple trait pairs including WD-SPAD, SPAD-SB, SPAD-V and LA-SPAD simultaneously. It was illustrated that lg17 was not only an important region related to leaf phenotype growth and development, but was also related to C allocation between SPAD and wood density.

The binary covariate genetic mapping analysis model was used to genetically analyse the unequal growth relationships between organs and phenotypes, in an attempt to better interpret the biological significance of *cov*QTLs (Gan et al., 2019). Various members in these gene families were involved in plant development, carboxylic acid metabolism, cell division and substance transportation. *RFC*4, *gag-pol*, integrase, and *KK1_037587* were involved in DNA replication and integration (Table 1; Supplementary Tables S2, S3). *RFC4* is a subunit of *RFC*, which might have a significant in *A. thaliana* embryo development, with embryonic lethality observed in *AtRFC* mutants (Zhang et al., 2020a). The *rglB* gene is involved in the carbohydrate metabolic process and catalytic activity. *CYCL1-1* is part of a molecular thermometer fine-tuning environmental information into the alternative splicing of target genes and is required for cell cycle regulation, transcription via RNA polymerase II and mRNA splicing (Cavallari et al., 2018; Nibau et al., 2019). Flagellin-sensitive 2 (*FLS2*), which was detected for many trait pairs, functions as a potent elicitor of the pathogen defence response in the cell wall and regulates receptor-mediated endocytosis as well as anion channel activity (Gomez-Gomez and Boller, 2000; Withers and Dong, 2017). The cytochrome P450 *CYP2* subfamily has been shown to participate in plant growth, namely in internode elongation by modulating the gibberellin response in rice (Luo et al., 2006) as well as cell elongation or growth in plant height via brassinosteroid biosynthesis (Hong et al., 2003; Chakrabarti et al., 2013).

We utilised transcriptomic data to identify a set of tissue-specific genes among those underlying C allocation as a complementary approach to QTL analysis. The genes derived from the overlap of differentially expressed genes and *cov*QTLs could be used to verify candidates. Two gene clusters were obtained, and the expression patterns in different tissues were in agreement with the *covQTL* distributions in trait pairs. Most of the highly expressed genes in leaves and petioles, which were low in the stems, were usually involved in the synthesis of biological macromolecules and cellular component organisation, including phospholipid binding and endocytosis, cellular amino acid biosynthetic process, DNA integration, exonuclease activity and methyltransferase activity (Figure 6; Supplementary Tables S2, S3). *GAD*/*SPL*, *TDX* and *CSTF64* were upregulated in all tissues except the xylem. *GAD*/*SPL* was the key gene related to five trait pairs and *cov*QLTs, being involved in the regulation of floral organ morphogenesis and formation as well as shoot apical meristem development (Wei et al., 2015). *SPL* interaction partner *YAB*3 was reported to sustain adaxial-abaxial polarity by specifying the abaxial cell fate (Fritz et al., 2018). Further, YAB genes participate in expanding flat leaf development through genetic programs that are related to marginal auxin flow and the activation of a maturation schedule directing determinate growth (Machida et al., 2015). *CSTF64* regulates developmental growth through mRNA surveillance, the antisense RNA metabolic process and regulation of gene silencing (Czesnick and Lenhard, 2016). *CLPT2*, which was upregulated only in leaves, participated in proteolysis protein metabolic processes and chloroplast organisation. The *CLPT1* and *CLPT2* proteins are unique in land plants to stabilise the Clp core complex (Moreno et al., 2017). In Arabidopsis, the *clpt1*/*clpt2* double mutant showed delayed growth, pale phenotype and altered leaf shape with more serrates on the leaf margins (Kim et al., 2015).

On the other hand, some genes were highly expressed in xylem and phloem instead of leaves and petioles. These were mainly responsible for material transportation. In particular, *COG1* regulates plant phototropism and gravitropism, participates in the detection of visible light as well as the response to light intensity and is involved in the far-red and red light signalling pathways, thus influencing the circadian rhythm of plants (Park et al., 2003). *COG1* may be of significance in light intensity detection in leaves as well as stem elongation. *MYB* plays a role in the negative regulation of gene expression, cellular macromolecule biosynthetic process and nitrogen compound metabolic process. In trees, the overexpression of *MYB* transcription factors results in enhanced photosynthesis, antioxidant enzyme production and enhanced growth under water stress (Polle et al., 2019). *ABC* plays a role in the biosynthetic process of dTMP, organonitrogen compound and organophosphate as well as in organic substance transmembrane transport (Supplementary Tables S2, S3). The *MTERF4* (*BSM*) detected in a significant QTL region for SPAD_SB presented a high expression level in both phloem and leaf, highlighting the pleiotropy of this gene. *MTERF4* participates in the regulation of mitotic cell cycle spindle assembly checkpoint as well as chloroplast organisation and is essential for normal plant development in addition to the maintenance of adequate levels of transcripts in both mitochondria and chloroplasts (Sun et al., 2016).

In summary, we explored the genetic basis of biomass allocation among organs using a bivariate QTL mapping model and verified the function of key genes via gene expression profiling in *C. bungei*, thus providing a basis for understanding the complex genetic regulation of trait-trait coordinated variation. The study of gene expression regulation further confirmed the reliability of *cov*QTLs obtained via the binary covariate genetic mapping model and helped explain the biological function of key genes. Narrowing down candidate genes could make functional verification feasible in future studies. Importantly, more biologically meaningful genes will be identified through multivariate (bivariate or even trivariate) mapping for multi-trait genetic dissection at different growth stages of perennial woody plants. Further, the integration of metabolomics, phenomics, genetic mapping, gene expression profiling, enzymatic activity analysis and genome-editing techniques will facilitate the identification of candidate genetic factors, thus benefiting tree breeding.

## Data Availability

The original contributions presented in the study are publicly available in NCBI using accession number PRJNA551333.
