# Supplementary material for: A Bivariate Mapping Model Identifies Major Covariation QTLs for Biomass Allocation Between Leaf and Stem Growth of Catalpa bungei
Source: Front Genet. 2021 Nov 18;12:758209. doi: 10.3389/fgene.2021.758209 (PMC8637733; doi:10.3389/fgene.2021.758209)
Supplement: Supplementary file 1 [file DataSheet1.docx]

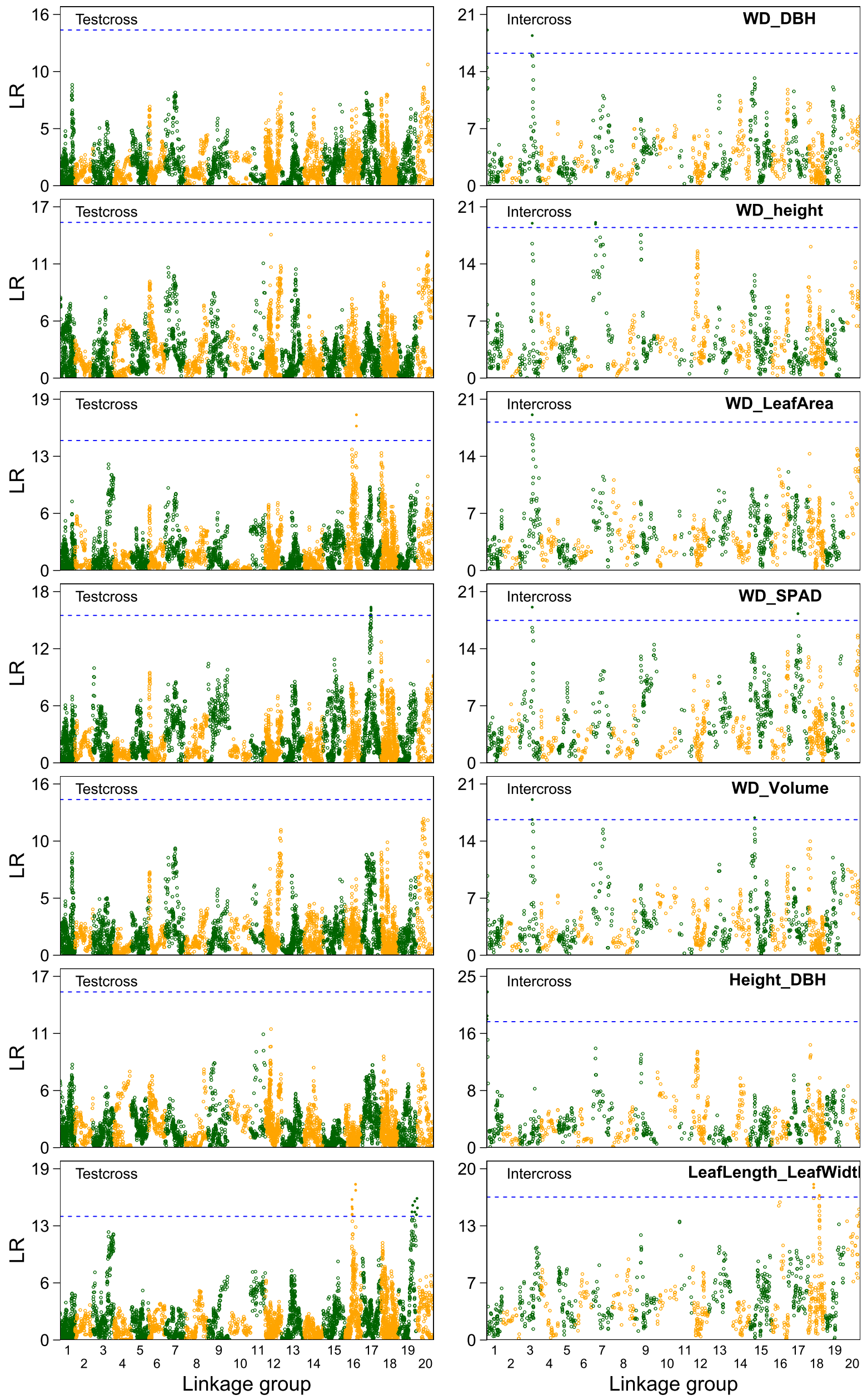


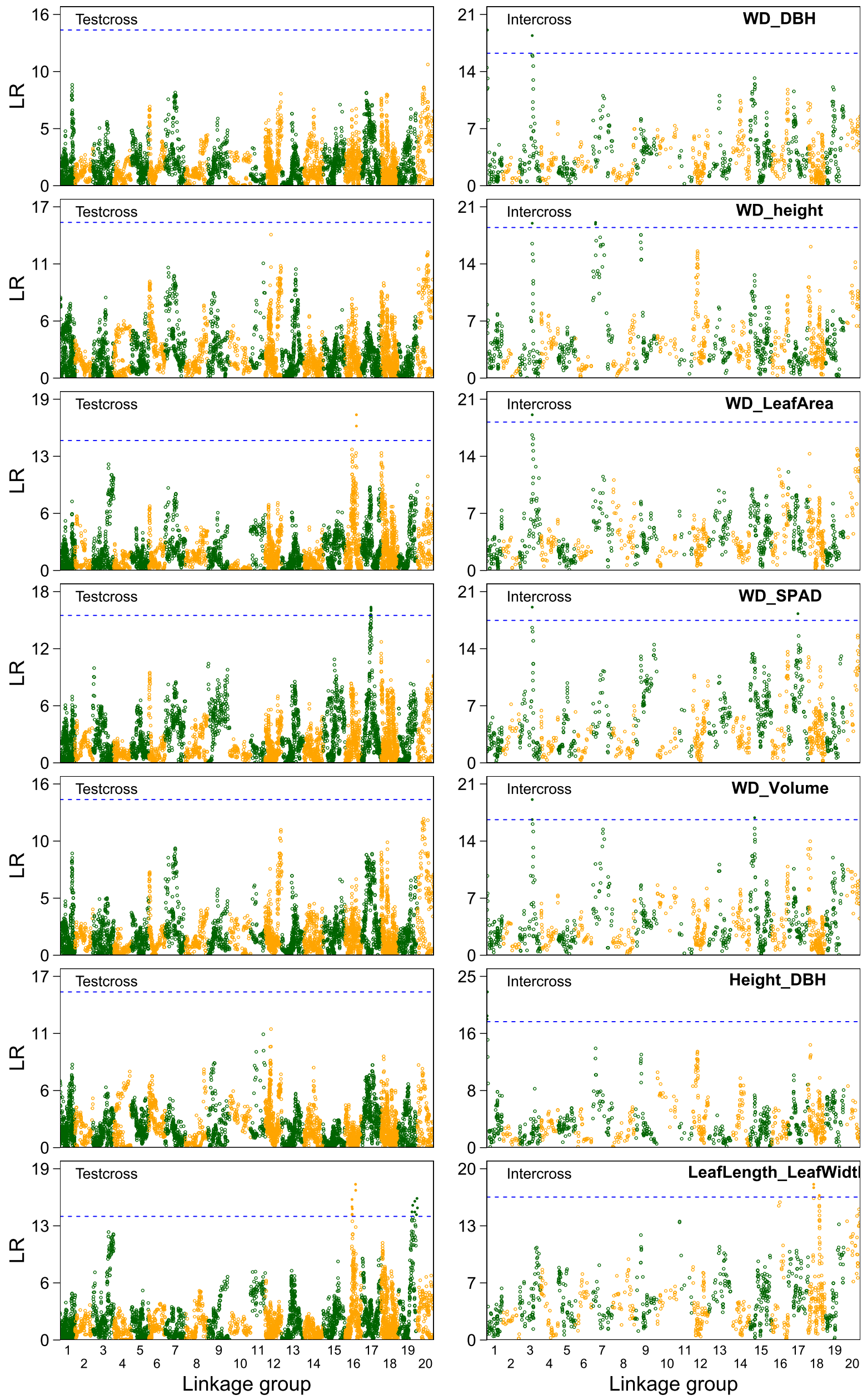


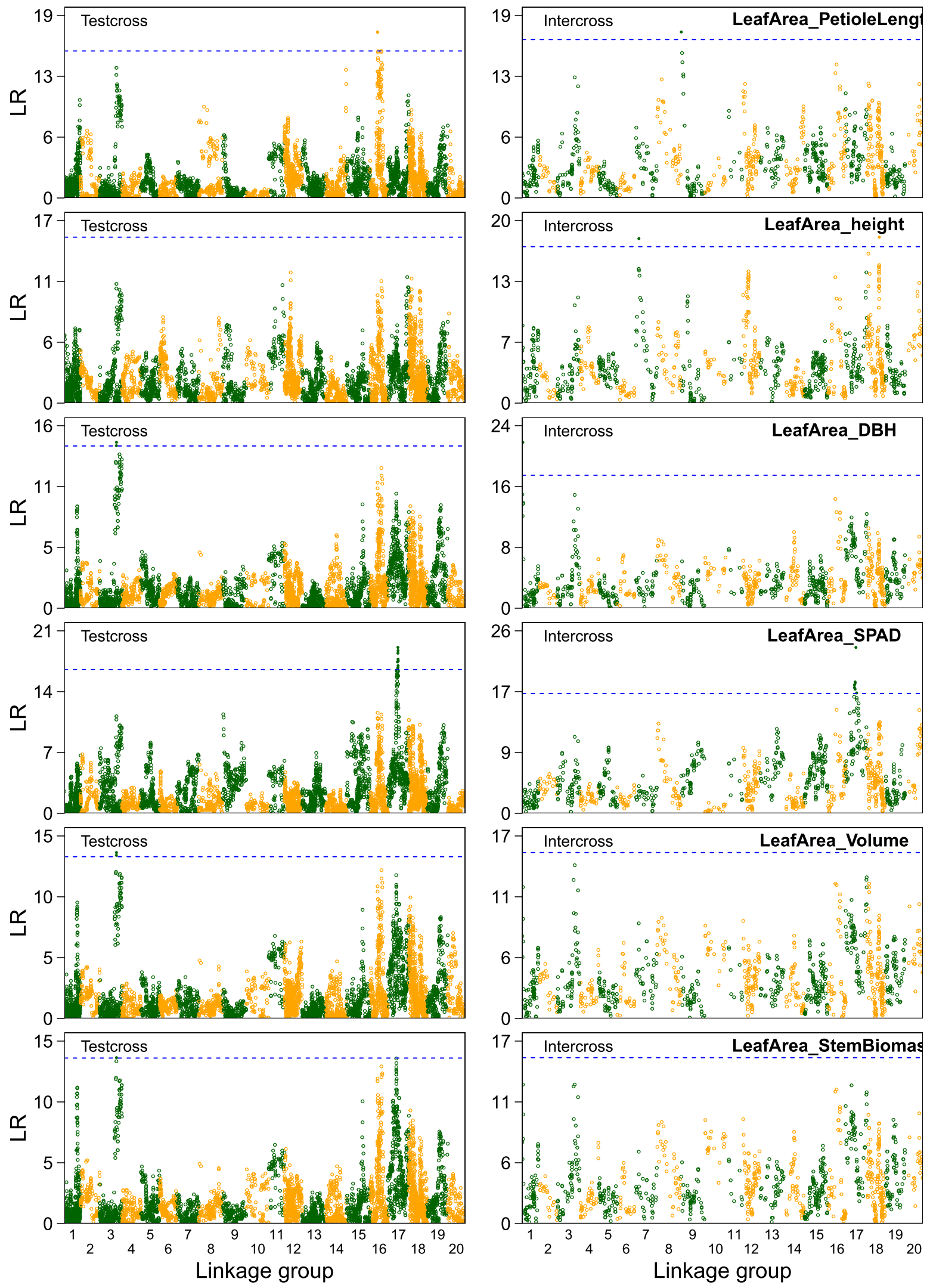


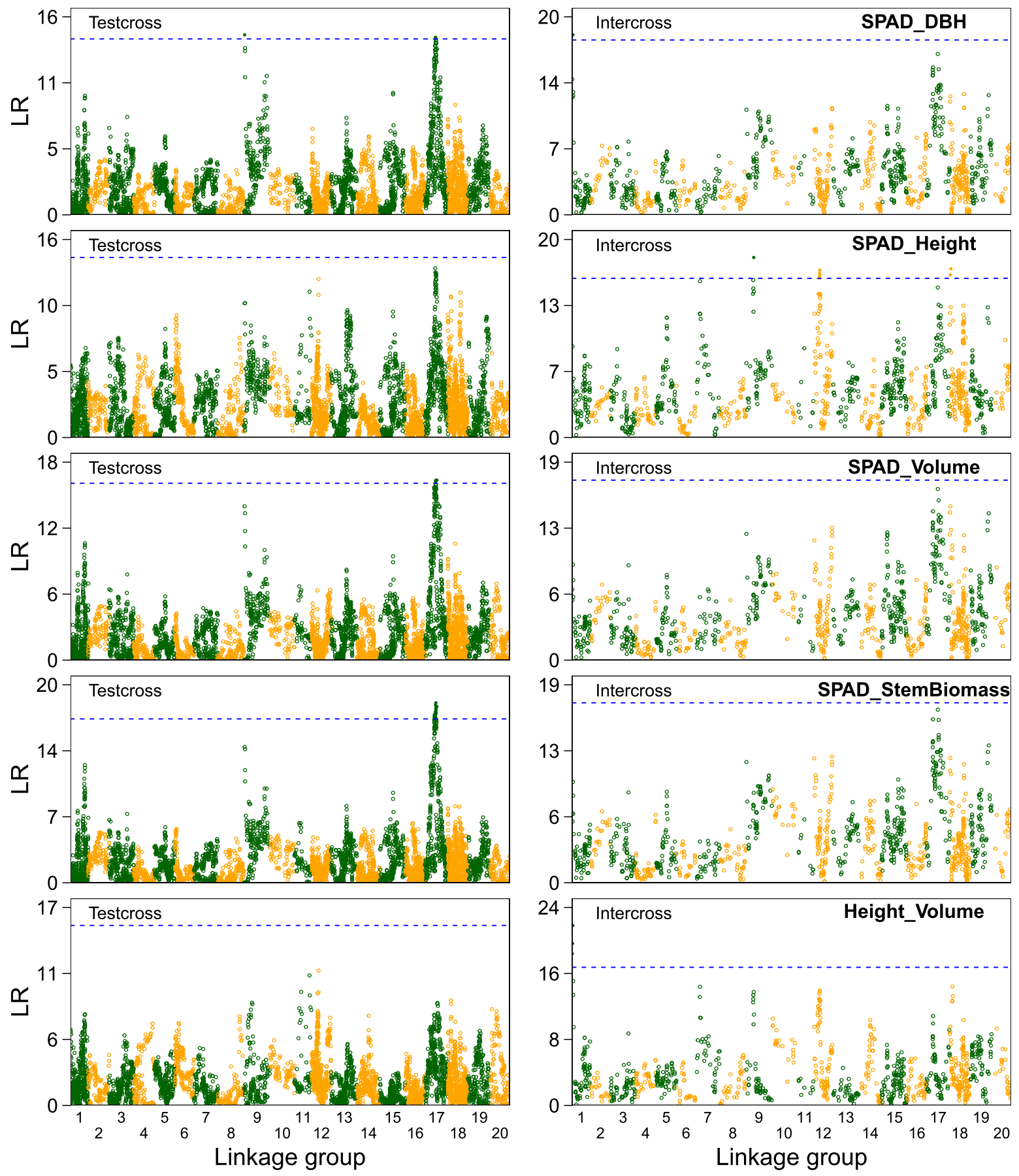


**Figure S1** Manhattan plots of significance tests for all testcross and intercross single nucleotide polymorphisms (SNPs) for 23 trait-trait pairs derived from six leaf traits (leaf length, width, area, perimeter, length/width ratio and petiole length) and five stem traits (height, diameter at breast height, wood density, stemwood volume and stemwood biomass). Blue dashed lines are the critical thresholds at the 5% significance level obtained via the permutation test.
